# Mesoporous Silica Derived from Municipal Solid Waste Incinerator (MSWI) Ash Slag: Synthesis, Characterization and Use as Supports for Au(III) Recovery

**DOI:** 10.3390/ma14226894

**Published:** 2021-11-15

**Authors:** Yosep Han, Seongsoo Han, Seongmin Kim, Minuk Jung, Ho-Seok Jeon, Siyoung Q. Choi, KyuHan Kim, Youngjae Kim

**Affiliations:** 1 Resources Recovery Research Center, Mineral Resources Division, Korea Institute of Geoscience & Mineral Resources (KIGAM), Daejeon 34132, Korea; smkim@kigam.re.kr (S.K.); mujung@kigam.re.kr (M.J.); hsjeon@kigam.re.kr (H.-S.J.); 2 Department of Resources Recycling, University of Science and Technology (UST), Daejeon 34113, Korea; 3 Department of Chemical and Biomolecular Engineering, Korea Advanced Institute of Science and Technology (KAIST), Daejeon 34141, Korea; sshan12@kaist.ac.kr; 4 Department of Chemical and Biomolecular Engineering, Seoul National University of Science and Technology, Seoul 01811, Korea

**Keywords:** MSWI ash slag, mesoporous silica, NaOH concentration, surface silanol, gold recovery

## Abstract

In this study, the effect of NaOH on the synthesis of mesoporous silica (MS) by using municipal solid-waste incinerator (MSWI) ash slag was investigated. Moreover, the prepared MS was used as a support to evaluate its potential for the recovery of gold ions (Au(III)) from aqueous solution. The extraction process for the MSWI ash slag activated through mechanical grinding entailed alkali treatment, using varying concentrations of NaOH. The content of Si extracted from MSWI ash slag increased with the increasing grinding time and NaOH concentration. As the NaOH concentration increased, the pore structure (e.g., Brunauer–Emmett–Teller (BET) surface area and pore volume) of the synthesized MS improved. In addition, the amount of adsorbed Au(III) increased with increasing sulfur content immobilized on the support, and the sulfur content was in turn governed by the silanol content of the MS support. The adsorbent prepared by using the MS-3M support exhibited the highest Au(III) adsorption capacity (110.3 mg/g), and its adsorption–desorption efficiency was not significantly affected even after five adsorption–desorption cycles.

## 1. Introduction

Mesoporous silica (MS) is a typical porous material with regularly arranged pores, several to tens of nanometers in size. This functional material is widely utilized as an adsorbent and catalyst support, and as a chemical, electrical and optical sensor [1,2,3,4,5,6,7]. Recent synthetic methods toward MS have been categorized as MCM (Mobil Composition of Matter)-type or SBA (Santa Barbara Amorphous material)-type, and tetraethyl orthosilicate (TEOS), fumed silica, water glass and sodium silicate are generally used as silica sources [8,9,10,11,12,13]. The use of these raw materials has the advantages of high purity, rapid reaction time and stable mesoporous material acquisition; however, TEOS is an expensive reagent, which in turn makes the product uneconomical [14,15,16]. Moreover, it is a toxic silica precursor and is difficult to handle [15,16]. Therefore, to compensate for these shortcomings, research is being actively conducted to achieve economic feasibility and implement recycling, whereby waste can be used as an alternative precursor material [14,15,16,17,18,19].

The amount of municipal solid-waste incinerator (MSWI) ash is rapidly increasing, and approximately 6 × 10^6^ tons of MSWI ash is generated annually in South Korea [20]. Although incineration has the advantage of reducing the overall weight and volume [21], MSWI ash is difficult to stabilize and treat adequately because of the concentration of harmful substances, such as dioxins and heavy metals [18,20].

Among the stabilization and recycling methods developed thus far, the melting method is the most stable and economical, because, although the operating cost is somewhat high, the volume of the final product is reduced by approximately 30–50%, which makes it the most effective method [20]. In addition, this method increases the temperature of the incineration ash to 1300–1400 °C to completely decompose harmful substances, and the heavy-metal component is significantly stabilized through the formation of a solid solution in the internal structure of the slag [18,20].

Among inorganic wastes, coal fly ash generally has pozzolanic properties and contains a large amount of silica (60–70%), alumina (16–20%) and trace amounts of transition metals. Therefore, studies on the synthesis of mesoporous materials using coal fly ash have been reported [22,23,24]. However, in the case of general incinerated ash slag, such as MSWI ash slag, which has undergone the melting process, most studies have focused on its use as a civil-engineering building material, despite its high silica and alumina content [25]. In the case of MSWI ash slag and coal fly ash, high aluminosilicate content is generated as a glassy substance; hence, they are being studied as a potential source of Al and Si [17,26]. However, MS synthesis by using incineration slag has rarely been performed.

In this study, MSWI ash slag, a waste resource, was treated with acid and mechanically activated, and then used as a starting material for the synthesis of MS via alkali treatment, using aqueous NaOH solution. We investigated the effect of NaOH concentration on the pore-related properties of the synthesized MS, particularly the BET surface area, pore size distribution and pore volume. Several complementary techniques, including TEM (transmission electron microscopy), nitrogen sorption isotherms and TGA (thermogravimetric analysis) analysis, were used to systemically evaluate the textural properties of the MS synthesized from MSWI ash slag.

Furthermore, the MS was functionalized with 3-mercaptopropyl-trimethoxysilane (3-MPTMS, -SH) via the impregnation method. In addition, the recovery of Au(III) present in an aqueous solution was evaluated by using -SH functionalized on the synthesized MS supports. Therefore, using MSWI ash slag, we attempted to identify the optimal conditions (NaOH concentration and grinding time) for the synthesis of MS. Accordingly, the possibility of using MSWI ash slag as a functional material for accessing mesoporous materials was evaluated.

## 2. Materials and Methods

### 2.1. Materials and Chemicals

The MSWI ash treated at 1300 °C, that is, MSWI ash slag, used as the starting material for synthesizing mesoporous silica (MS), was collected from South Korea. The surfactant, P123 (EO_20_PO_70_EO_20_, M_w_ = 5800, 30 wt.%), 3-mercaptopropyl-trimethoxysilane (3-MPTMS, 95%), sulfuric acid (H_2_SO_4_, 2 M), hydrochloric acid (HCl, 37%), toluene (99.8%), sodium hydroxide (NaOH, >98%), thiourea (>99%) and gold(III) chloride (99%) were obtained from Sigma-Aldrich. The deionized water used in all the experiments was prepared by using a three-stage Millipore Mill-Q Plus 185 purification system.

### 2.2. Synthesis of MS and Thiol-Functionalized MS from MSWI Ash Slag

In this study, the MS support synthesis process from MSWI ash slag is shown in Figure S1 of Supplementary Materials. First, to remove the various impurities contained in the MSWI ash slag, an acid pretreatment was performed. Thus, MSWI ash-slag powder and 2 M H_2_SO_4_ were mixed in a ratio of 1:10 and reacted at 80 °C with stirring at 100 rpm for 24 h, after which the supernatant and solid were separated. Thereafter, the solid was dried at 80 °C for 24 h to obtain the acid-treated MSWI ash slag as a powder. The dried powder was then mechanically activated by using a dry ball mill to obtain pretreated fine MSWI ash slag. Alumina balls with a diameter of 5 mm were used as the grinding media, and the filling ratio of grinding media to sample was 4:3. The rotational speed of the ball mill was 120 rpm.

Si ions were extracted from the pretreated fine MSWI ash slag, using aqueous NaOH in a concentration range of 1–4 M. To 500 mL of each NaOH solution was added 30 g of pretreated MSWI ash-slag powder and the suspension was stirred at 200 rpm and 90 °C for 24 h. Thereafter, the precipitate was removed by suction filtration to obtain the Si extract.

To synthesize MS using the Si extract, 4 g of P123 was dissolved in 120 mL of 2 M HCl by stirring at 35 °C. Then, 46 g of the Si extract was added to the above solution, followed by stirring at 35 °C for 24 h and ageing at 90 °C for 24 h. The synthesized solid was collected by filtration and stirred in ethanol for 24 h to remove the surfactant and obtain MS (see Figure S1). The obtained MS samples were referred to as MS-XM, where X = 1, 2, 3 or 4, corresponding to the molar concentration of NaOH used in the synthesis.

To immobilize –SH groups on MS [27], 1 g of MPTMS dispersed in 100 mL of toluene was added to 1 g of MS, and the mixture was heated at reflux for 24 h. Thereafter it was filtered to obtain a solid, which was washed thoroughly with ethanol and toluene and dried at 60 °C for 24 h. The thiol-functionalized MS prepared from MSWI ash slag is referred to as MS-XM-SH (thiol-functionalized MS prepared using 1, 2, 3 or 4 M NaOH).

### 2.3. Characterization

Powder X-ray diffraction (XRD) patterns were obtained by using a Bruker D8 HRXRD X-ray diffractometer (D8, Bruker, Billerica, MA, USA) with Ni-filtered CuKα radiation (λ = 0.154606 nm, 40 kV, 40 mV). Nitrogen adsorption–desorption isotherms were obtained using a physisorption analyzer (3Flex, Micromeritics, Atlanta, GA, USA) at −195 °C. The specific surface area was then calculated from a relative pressure (P/P_O_) range of 0.05–0.25 employing the Brunauer–Emmett–Teller (BET) method [28,29]. The mesopore volume and size distribution were determined from the nitrogen adsorption branch, and the mesopore size distribution was calculated by using the Barrett–Joyner–Halenda (BJH) method [30,31]. The TEM (JEOL-2100F, JEOL, Tokyo, Japan) samples were suspended in ethanol and dropped onto holey carbon films supported on Cu grids for imaging. Thermogravimetric measurements were performed by using a thermobalance (TGA N-1000, Scinco, Seoul, Korea) with standard platinum crucibles and 10 mg of sample. A procedure developed by Mueller et al. and Peng et al. was adapted to determine the surface silanol density and content of the prepared MS supports [32,33]. The heating profiles were set as follows under a nitrogen atmosphere: the samples were stabilized at 20 °C for 5 min and then heated at a ramp of 10 °C/min to a 200 °C isotherm for 30 min. The materials were then heated to 800 °C at a ramp of 20 °C/min. The surface silanol density (SD/nm^2^) was calculated based on the weight loss (g) occurring between 200 and 800 °C, using Equation (1):

(1) $$\mathrm{SD}=\alpha \left(\frac{{\mathrm{wt}}_{T_{1}}-{\mathrm{wt}}_{T_{2}}}{{\mathrm{wt}}_{T_{1}}}\right)\frac{2N_{A}}{{\mathrm{SA}}_{\mathrm{BET}}\times \mathrm{Mw}}$$

where α = 0.625 (calibration factor), T_1_ = 200 °C, T_2_ = 800 °C, N_A_ is Avogadro’s number, SA_BET_ (nm^2^/g) is the BET surface area and Mw is the molecular weight of water. Surface silanol content (SC/g) was determined by using Equation (2):

(2) $$\mathrm{SC}=\mathrm{SD}\times {\mathrm{SA}}_{\mathrm{BET}}$$

The sulfur content was quantified by using an elemental analyzer (Flash EA 1112, Thermo Finnigan, San Jose, CA, USA).

### 2.4. Au(III) Recovery Performance

The Au(III) adsorption capacities of thiol-functionalized MS samples were evaluated by adding 0.1 g of the adsorbent to 100 mL of aqueous Au(III) (300 mg/L). The pH of the aqueous solution was adjusted to 2.0 by adding the appropriate amount of HCl and NaOH to maximize Au(III) ion recovery [27]. The adsorption experiments were performed in batches, stirring at 200 rpm and 25 °C for 24 h. The suspension was filtered to remove the sorbent, and the residual concentration of Au(III) in the filtrate was measured by using an inductively coupled plasma atomic emission spectrometer (ICP–AES, Vista Pro, Varian, Palo Alto, USA). The Au(III) adsorption capacity (Qe) of thiol-MS was calculated by using Equation (3) [27,34]:

(3) $$Q_{e}=\frac{C_{i}-C_{e}}{m}\times V$$

where Qe (mg/g) is the amount of solute adsorbed per unit mass of adsorbent; Ci (mg/L) and Ce (mg/L) are the initial and equilibrium concentrations of the solute in solution, respectively; m (g) is the weight of the sample; and V (L) is the volume of the aqueous solution.

After the adsorption experiments, Au(III) ion-adsorbed thiol-functionalized MS was separated from the aqueous solution by filtration and centrifugation. Then, desorption of the samples was achieved by stirring 0.1 g of used adsorbent in 1 L of 2 M HNO_3_ and 0.1 g thiourea at 200 rpm and 25 °C for 24 h. This procedure was repeated five times, and the adsorption capacity was measured after each cycle.

## 3. Results and Discussion

### 3.1. Synthesis of Mesoporous Silica from MSWI Ash Slag

Table 1 lists the chemical composition of the MSWI ash slag used. The chemical analysis revealed that the SiO_2_ component required to prepare MS accounted for 57 wt.% of the total content, and the Al_2_O_3_ content was 14 wt.%.

**Figure 1:**
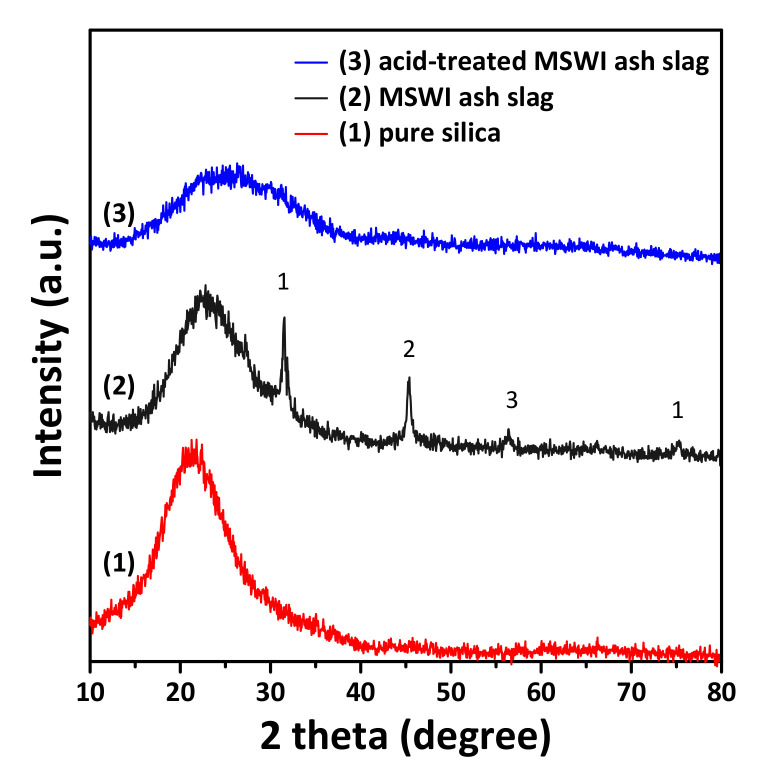
XRD patterns of MSWI ash slag used as the starting material, treated MSWI ash slag obtained after acid treatment, and pure silica (for comparison): (1) gehlenite (Ca_2_Al_2_SiO_7_), (2) magnetite (Fe_3_O_4_) and (3) hematite (Fe_2_O_3_).

Figure 1 shows the XRD patterns of pure silica, MSWI ash slag and acid-treated MSWI ash slag. The XRD pattern of the MSWI ash slag used in this study was similar to that of silica, except that the former displayed peaks corresponding to gehlenite, magnetite and hematite. As shown in Figure 1, the silica peak intensity of the acid-treated incinerated ash slag decreased relative to that of MSWI ash slag, and the peaks (i.e., gehlenite, magnetite and hematite) did not appear. This indicates that the acid treatment successfully removed the metallic impurities from the MSWI ash slag, and it is considered that alkali-based metal oxides, including silica, which are the main components, remained. 

**Figure 2:**
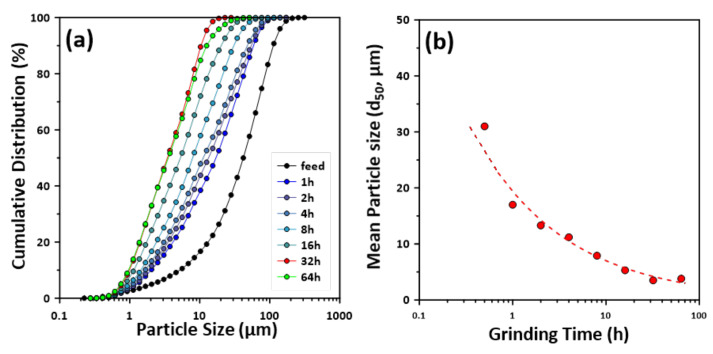
(**a**) Particle size distribution and (**b**) mean particle size of the MSWI ash slag as a function of grinding time. Mean particle size (d_50_) of the raw acid-treated MSWI ash slag used in this study was 42.5 µm.

The particle size distribution and mean particle size (d_50_) of the acid-treated MSWI ash slag milled by using a ball mill are shown in Figure 2. The particle size distribution of the feed, that is, the raw acid-treated MSWI ash slag, was 0.5–250 µm and the mean particle size was 42.5 µm. As the grinding time increased, the particle size distribution and mean particle size decreased. However, the mean particle size increased to approximately 3.8 µm at 64 h compared to approximately 3.5 µm at a grinding time of 32 h. It is considered that, at a grinding time of ≥64 h, no further particle reduction is observed, as agglomeration occurs due to the grinding process. In general, when grinding in dry conditions, the surface energy of fine particles (several µm) increases considerably, which facilitates agglomeration, and, therefore, the particle size increases [35]. Based on these results, it was surmised that the mechanical activation effect for the acid-treated MSWI ash slag would be maximized at a grinding time of 32 h.

**Figure 3:**
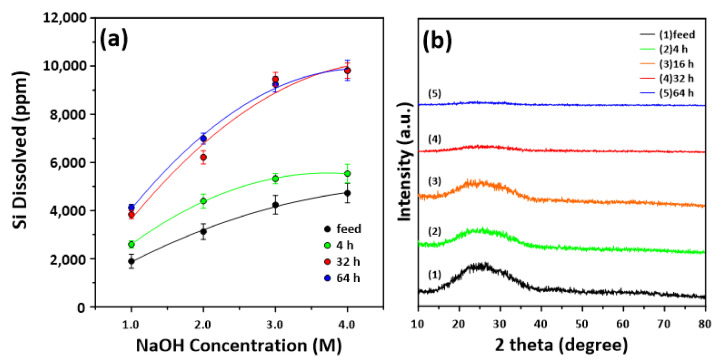
(**a**) Si dissolution from raw MSWI ash slag (feed) and the activated MSWI ash slag milled at 4, 32 and 64 h as a function of NaOH concentration. (**b**) XRD patterns of raw MSWI ash slag and activated MSWI ash slag milled at 4, 16, 32 and 64 h.

In general, because inorganic-based waste resources are used as starting materials for MS, it is crucial to achieve excellent silica yields [17,26]. Therefore, we analyzed the amount of Si ions extracted from the mechanically activated MSWI ash slag, using varying NaOH concentrations. Figure 3a shows the dissolved Si concentrations achieved at each NaOH concentration. Overall, the amount of Si extracted increased with increasing NaOH concentration, as well as with increasing grinding time. In the case of the feed sample, the Si concentration increased significantly from 1 to 3 M NaOH, but it increased relatively little at 4 M. The amounts of silica extracted from the MSWI ash slag milled for 64 h at 3 and 4 M were 9242 and 9823 ppm (the highest), respectively. In all the milled MSWI ash slags, the amount of Si extract increased linearly from 1 to 3 M NaOH, with a relatively slight increase at 4 M. Therefore, it was determined that a NaOH concentration of ≥ 4 M did not significantly affect the yield. In addition, the amount of extracted Si was compared for the ash slag samples before and after acid treatment employing ICP analysis. For the non-acid-treated MSWI ash slag, at a grinding time of 64 h, the extracted Si concentrations at 1–4 M NaOH were 1472, 2860, 4780 and 4174 ppm, respectively, being significantly lower than that extracted from the acid-treated MSWI ash slag.

In general, mechanical activation through grinding changes the surface energy of a target solid material through chemical conversion, such as fracture, amorphization and chemical reactions [35,36]. The energy generated during grinding accumulates on the surface and inside the particle, causing lattice strain and various chemical-phase transformations [35,36]. Therefore, in this study, the XRD patterns of MSWI ash slag activated by grinding at varying times were obtained, as shown in Figure 3b. As the grinding time increased, the major XRD peak decreased, being almost invisible after grinding for 32 h. Therefore, the MSWI ash slag milled for ≥32 h was crystalline and was converted to an amorphous state with a regular structure. Based on these results, it was concluded that the MSWI ash slag was converted to an amorphous state after 32 h of mechanical treatment, and that the amount of Si extracted increased significantly when the extraction process was performed by using NaOH. Accordingly, the pulverization time of MSWI ash slag was set to 32 h, and MS was synthesized by using various concentrations of NaOH.

### 3.2. Characterization of the Synthesized Mesoporous Silica: Effect of NaOH Concentrations

**Figure 4:**
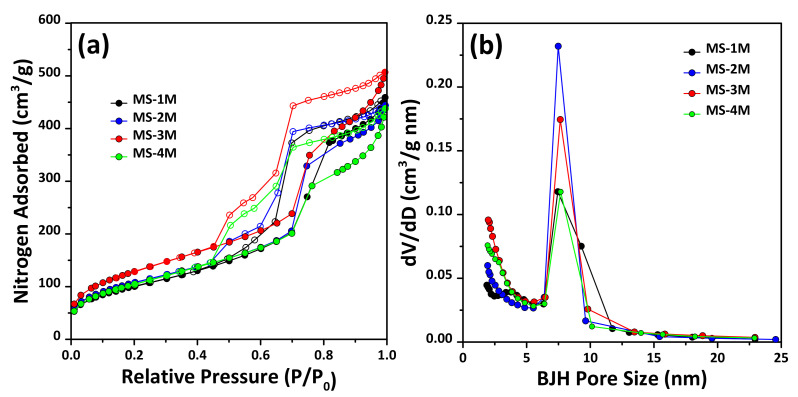
(**a**) Nitrogen adsorption–desorption isotherms and (**b**) pore size distributions of MS-1M, MS-2M, MS-3M and MS-4M, respectively. The isotherms have not been offset. Solid circle and empty circle symbol represent adsorption and desorption curve, respectively. Pore size distributions derived by using BJH mesopore analysis from the nitrogen adsorption isotherms are presented in (**a**).

Figure 4 shows the nitrogen adsorption–desorption isotherms and mesopore size distributions of MS synthesized by using varying NaOH concentrations. Overall, the nitrogen adsorption–desorption isotherms of all the samples corresponded to type IV with an H1 hysteresis loop, according to the IUPAC classification [30,37]. This indicates that all the synthesized samples consisted predominantly of mesopores, as the hysteresis point was observed at P/Po = 0.45 [30,37].

The mesopore size distributions of the samples were measured, as shown in Figure 4b, and are summarized in Table 2. The pore size was found to be approximately 7 nm within the NaOH concentration range controlled in this study. Therefore, it was found that the NaOH concentration had no effect on the pore size of the meso-structure.

**Figure 5:**
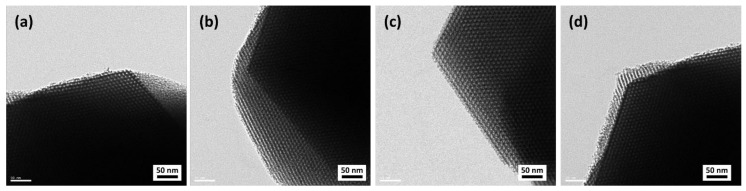
TEM images of the MS samples synthesized from MSWI ash slag at various NaOH concentrations: (**a**) MS-1M, (**b**) MS-2M, (**c**) MS-3M and (**d**) MS-4M.

In addition, we attempted to confirm the meso-structure of the prepared samples employing TEM analysis, and the results are shown in Figure 5. Overall, as the hexagonal pore structure was observed in all the samples, it was confirmed that they were prepared as typical SBA-15 MS [7,27,30,37]. According to the pore size distribution, it was confirmed that meso-structures with a pore size of approximately 7 nm were mainly formed regardless of the NaOH concentration. However, as the concentration of NaOH increased from MS-1M to MS-3M, the BET specific surface area and total pore volume increased slightly, but decreased in the case of MS-4M.

Accordingly, it was observed that the meso-structure of MS was well formed as the NaOH concentration increased, but at 4 M, the highest concentration of NaOH, as the BET specific surface area and total pore volume decreased, the formation of the meso-structure was no longer dominant. Two possible rationales can explain the increase up to 3 M NaOH, followed by a decrease at a high concentration of 4 M. First, to investigate the influence of the Si/Al ratio of all the MS samples, the Si/Al ratio was determined by employing ICP analysis, and the results are shown in Table 2.

In this study, as the NaOH concentration increased, the amount of Si extracted from the MSWI ash slag increased, while the Al concentration remained virtually constant. Therefore, the Si/Al ratio increased until the NaOH concentration was 3 M, and the BET specific surface area and pore volume increased as the Si/Al ratio increased. This was consistent with previous studies [38], and previously reported ^29^Al NMR and XPS results have shown that as the Al concentration increased compared to that of Si, the formation of Al_x_O_y_ occurred, resulting in deformation of the pore size of the synthesized MS. Thus, it is considered that the BET specific surface area and total pore volume of the synthesized MS increased as the content of Si leached from the MSWI ash slag increased. However, in MS-4M, which had the highest Si/Al ratio, the BET specific surface area and total pore volume decreased.

According to the results of a study on the synthesis of MS from waste resources and natural minerals, such as fly ash, bottom ash and anorthite–clay [6,8,11,14,36], it was reported that Na ions coexisting with NaOH interfered with the formation of mesophase due to the high NaOH concentration [39]. Based on these results, it was surmised that the pore structure of MS-4M, which was the highest yielding Si product, deteriorated compared to that of MS-3M, as the formation of mesophase was hindered by the presence of a relatively high excess of Na ions at 4 M NaOH. Therefore, a NaOH concentration of 3 M is considered optimal for the formation of a favorable pore structure (based on BET specific surface area and pore volume) when synthesizing MS from the MSWI ash slag used in this study.

**Figure 6:**
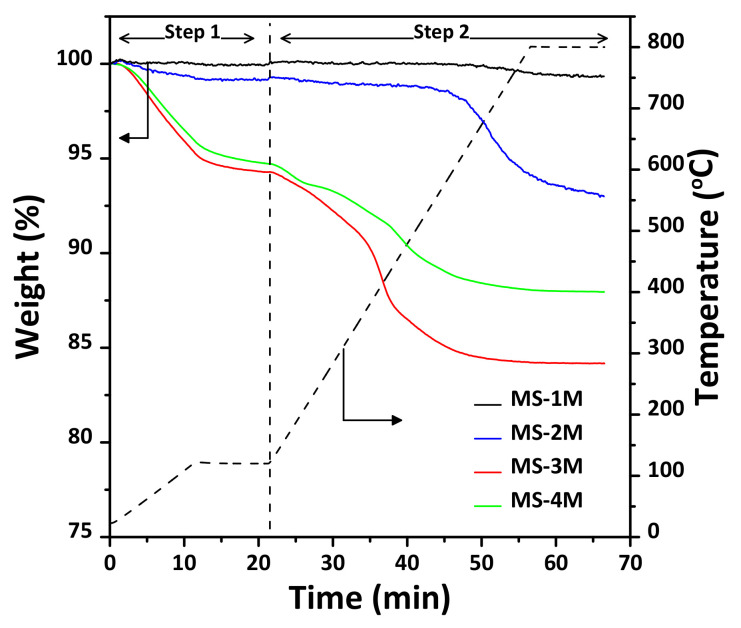
TGA profiles for the MS synthesized by using varying NaOH concentrations. Normalized weight of the mesoporous silica and TGA temperature (black dash line) as a function of TGA heating time. The first step (Step 1) removes the physically adsorbed water. The surface Si-OH density and content (i.e., chemisorption water) is based on the weight loss of Step 2.

To quantitatively analyze the surface silanol content of the synthesized MS supports, TGA analysis was performed. Figure 6 shows the TGA profiles of MS synthesized by using varying NaOH concentrations and the MSWI ash slag. It is well-known that both physically bound or hydrogen-bonded water and silanol groups exist on the surface of silica, but are anchored with distinct bonds [33,34]. Therefore, according to previous reports, silica undergoes dehydration of surface water differently at 120 and 800 °C. Interestingly, the silanol concentration increased with increasing NaOH concentration until 3 M, being the highest in MS-3M (see Table 2), whereupon it decreased in the MS-4M sample. It is likely that the excess Na ions affected the formation of silanol on the silica surface. Therefore, it is necessary to evaluate the silanol properties (concentration and adsorption state, etc.) formed on the surface of MSWI ash slag in the future.

The thiol functional group (-SH) was immobilized by using MS supports prepared by using varying NaOH concentrations to evaluate its potential as an adsorbent for Au(III) recovery in aqueous media. Accordingly, the sulfur concentration was evaluated to determine the concentration of -SH adsorbed [6,27]. A difference in the sulfur concentration compared to the silanol concentration was observed, and the sulfur content increased as the silanol concentration increased. In particular, a high sulfur concentration of 3.3% was observed in the MS-3M support, which had the highest silanol content. Therefore, it was concluded that the presence of surface silanol groups increases the sulfur content on the surface of the MS supports.

### 3.3. Recovery Performance of Au(III) in Aqueous Solution

**Figure 7:**
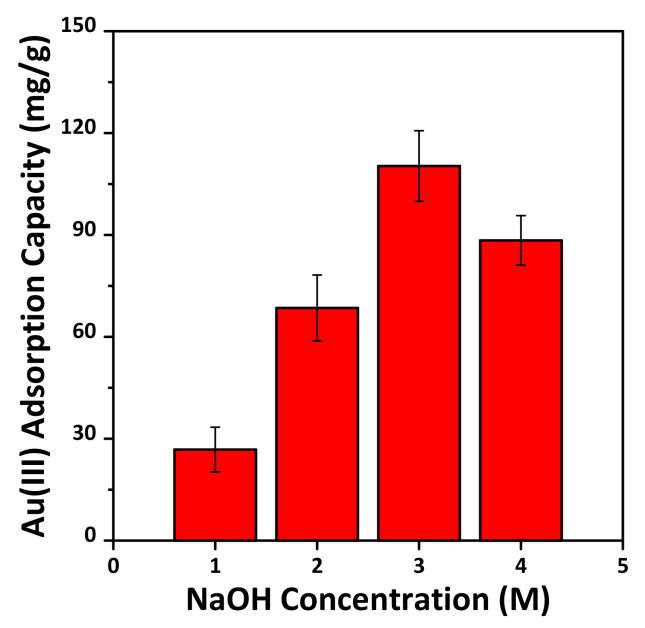
Maximum Au(III) adsorption capacity of the thiol-functionalized samples, MS-M1-SH, MS-M2-SH, MS-M3-SH and MS-M4-SH: pH 2.0, V/m = 1.0 L/g, [Au(III)] = 300 mg/L and 25 °C for 24 h. Error bars denote standard deviations.

As is well-known, research on the recovery of Au(III) present in a trace amount in aqueous solution has been reported on cost-competitive methods through various methods [27,40,41]. In this study, functional materials were manufactured from waste resources and the possibility of recovery of Au(II) in aqueous solution was conducted. Figure 7 shows the batch tests comparing the adsorption capacity of the -SH functionalized adsorbents, that is, the adsorbents prepared by immobilizing -SH on MS-M1 -MS-M4. Interestingly, a difference in the Au(III) adsorption performance was observed depending on the sulfur content immobilized on the support made of the MSWI ash slag used in this study. In particular, the maximum Au(III) adsorption capacity was 110.3 mg/g, achieved by using the MS-3M-SH adsorbent. This is in accordance with a recently reported result [27], whereby the high silanol content of the support not only increases the sulfur content but also promotes uniformity. Therefore, in this study, it was determined that the adsorption of Au(III) ions from aqueous solution was highest for the adsorbent with the highest sulfur content, generated from the MS-3M support having the highest silanol content.

**Figure 8:**
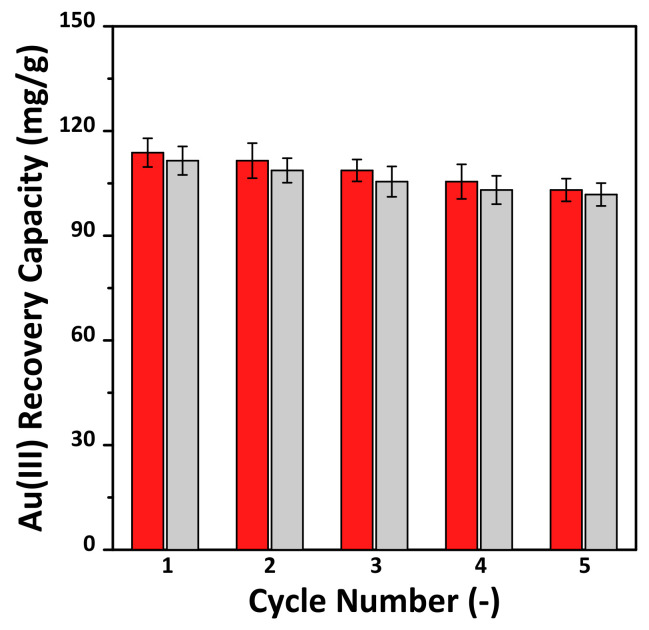
Cycle performance of Au(III) recovery capacity on the thiol-functional adsorbent (MS-3M-SH) prepared by using the synthesized MS-3M support: pH 2.0, V/m = 1.0 L/g, [Au(III)] = 300 mg/L and 25 °C for 24 h. Desorption was conducted at 2M HNO_3_ and 0.1 g thiourea solution at 25 °C for 24 h. Error bars denote standard deviations.

A good adsorbent should be able to be regenerated and its adsorption sites renewed for many cycles. The reusability can reduce the overall cost of the process and thus makes it more suitable for industrial applications [25,35,42,43,44,45]. We performed adsorption–desorption repetition experiments for five cycles for stable Au(III) recovery, using MS-3M-SH, and the results are shown in Figure 8. It was confirmed that the adsorption performance did not decrease significantly even after five cycles, and in particular, the adsorption capacity after five cycles was confirmed to be approximately 88% from the basis of one cycle. In addition, the desorption efficiency remained within approximately 97.5% of the adsorption, regardless of the number of cycles, compared to the adsorption capacity. These results demonstrate that the adsorbent synthesized from the MS-3M support enables effective and stable recovery of Au(III) from aqueous media.

## 4. Conclusions

In this study, MSWI ash slag was used as a starting material for the synthesis of MS. In particular, by acid treatment and mechanical activation, Si extraction characteristics according to various NaOH concentrations were identified. In addition, the possibility of recovering Au(III) from an aqueous solution was evaluated, and the results are as follows:

1. The amount of Si extracted was affected by the NaOH concentration, and as the grinding time of the MSWI ash slag increased, the Si extracted amount increased.
2. The MS synthesized from the MSWI ash slag was confirmed to be typical SBA-15 as MS having a hexagonal structure with an average pore size of 7 nm, regardless of the concentration of NaOH. While the BET specific surface area and pore volume of synthesized MS increased with increasing NaOH concentration, MS-4M, synthesized from an excessive concentration of 4 M, exhibited decreased BET specific surface area and total pore volume.
3. Regarding Au(III) recovery, the amount of adsorbed Au(III) was highest for the sample with the highest the sulfur content. The sulfur content was governed by the silanol content of the support. The MS-3M-SH adsorbent exhibited the greatest Au(III) adsorption capacity (110.3 mg/g), and its adsorption–desorption efficiency was not significantly affected even after five adsorption–desorption cycles.

Based on these experimental results, MSWI ash slag is expected to be used as an adsorbent for effective Au(III) recovery from aqueous media and as a sustainable, economical and environmentally friendly starting material for MS synthesis.

## Figures and Tables

**Table 1:** Chemical composition of the MSWI ash slag used in this study.

Material	Chemical Composition (wt.%)						
MSWI ash slag	SiO_2_	Na_2_O	P_2_O_5_	K_2_O	Al_2_O_3_	Fe_2_O_3_	CaO
	57.12	2.8	1.4	2.54	14.12	10.31	6.02
	TiO_2_	CuO	ZnO	Rb_2_O	MgO	Ig-loss	
	0.74	0.29	0.16	1.20	1.04	2.26	

**Table 2:** Physical properties, silanol (Si-OH) and sulfur content of the synthesized MS obtained in this study.

Sample (Support/Adsorbent) df	Si/Al ^1^	S_BET_ (m^2^/g)	Pore Volume (cm^3^/g)	Pore Size (nm)	SC (×10^21^/g) ^2^	S Content (%) ^3^
MS-1M/MS-1M-SH	28.5	368	0.709	7.44	0.44	1.2
MS-2M/MS-2M-SH	44.5	393	0.880	7.48	2.41	2.1
MS-3M/MS-3M-SH	60.1	471	0.998	7.64	4.91	3.3
MS-4M/MS-4M-SH	62.5	383	0.834	7.67	3.51	2.6

^1^ Si/Al ratio determined using ICP analysis. ^2^ Silanol contents (SC) of the MS supports (MS-XM) were analyzed by using TGA. ^3^ Sulfur (S) contents of the thiol-modified MS adsorbents (MS-XM-SH) were analyzed by using EA.

## Data Availability

Not applicable.

## Supplementary Materials

The following are available online at https://www.mdpi.com/article/10.3390/ma14226894/s1, Figure S1: The synthesis process flow diagram of mesoporous silica from MSWI ash slag.
