# Supplementary material for: Mesoporous Silica Derived from Municipal Solid Waste Incinerator (MSWI) Ash Slag: Synthesis, Characterization and Use as Supports for Au(III) Recovery
Source: Materials (Basel). 2021 Nov 15;14(22):6894. doi: 10.3390/ma14226894 (PMC8620291; doi:10.3390/ma14226894)
Supplement: Supplementary file 1 [file materials-14-06894-s001.zip › materials-1458813-supplementary.pdf]

Supplementary Materials

Mesoporous Silica Derived from Municipal Solid Waste Incinerator (MSWI) Ash Slag: Synthesis, Characterization and Use as Supports for Au(III) Recovery

Yosep Han [1,2,*†], Seongsoo Han [3†], Seongmin Kim [1], Minuk Jung [1], Ho-Seok Jeon [1,2], Siyoung Q. Choi [3,*], KyuHan Kim [4,*] and Youngjae Kim [1,2,*]

1 Resources Recovery Research Center, Mineral Resources Division, Korea Institute of Geoscience & Mineral Resources (KIGAM), Daejeon 34132, Korea; smkim@kigam.re.kr (S.K.); mujung@kigam.re.kr (M.J.); hsjeon@kigam.re.kr (H.-S.J.)
2 Department of Resources Recycling, University of Science and Technology (UST), Daejeon 34113, Korea
3 Department of Chemical and Biomolecular Engineering, Korea Advanced Institute of Science and Technology (KAIST), 291, Daejeon 34141, Korea; sshan12@kaist.ac.kr
4 Department of Chemical and Biomolecular Engineering, Seoul National University of Science and Technology, Seoul 01811, Korea
* Correspondence: yosep@kigam.re.kr, Tel.: +81-42-868-3181 (Y.H.); sqchoi@kaist.ac.kr, Tel. +92-42-350-3914 (S.Q.C.); youngjae.kim@kigam.re.kr, Tel. +82-42-868-3265 (Y.K.); kyuhankim@seoultech.ac.kr (K.K.)
† These authors contributed equally to this work

Citation: Han, Y.; Han, S.; Kim, S.; Jung, M.; Jeon, H.-S.; Choi, S.Q.; Kim, K.; Kim, Y. Mesoporous Silica Derived from Municipal Solid WasteIncinerator (MSWI) Ash Slag: Synthesis, Characterization and Use as Supports for Au(III) Recovery. *Materials* **2021**, *14*, 6894. https://doi.org/10.3390/ma14226894

Academic Editor: José Miguel Hidalgo Herrador

Received: 27 October 2021
Accepted: 9 November 2021
Published: 15 November 2021

Publisher's Note: MDPI stays neutral with regard to jurisdictional claims in published maps and institutional affiliations.

Copyright: © 2021 by the authors. Submitted for possible open access publication under the terms and conditions of the Creative Commons Attribution (CC BY) license (http://creativecommons.org/licenses/by/4.0/).

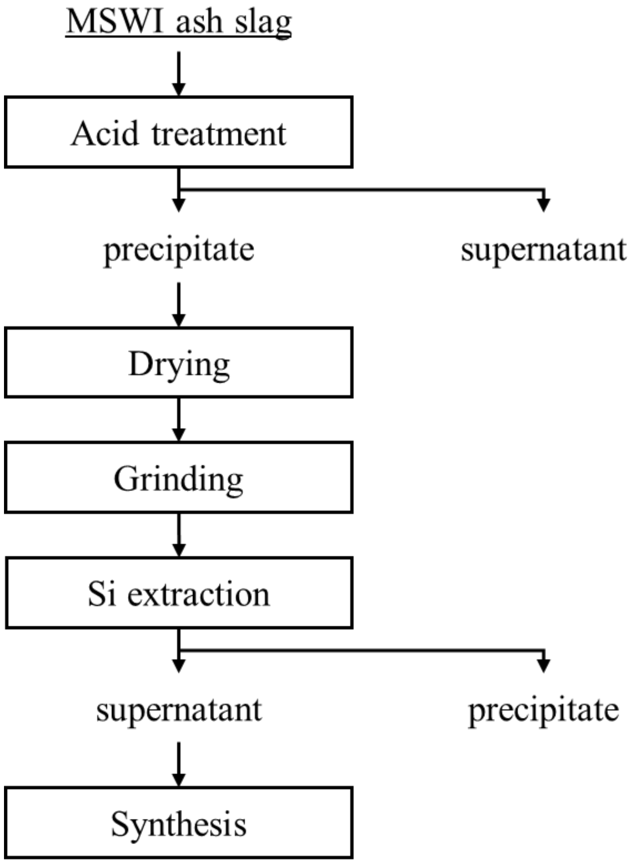

Figure 1. The synthesis process flow diagram of mesoporous silica from MSWI ash slag.
